# Supplementary material for: Low temperature synthesis of BiFeO3 nanoparticles with enhanced magnetization and promising photocatalytic performance in dye degradation and hydrogen evolution
Source: RSC Adv. 2018 Aug 21;8(52):29613–27. doi: 10.1039/c8ra04599b (PMC9085277; doi:10.1039/c8ra04599b)
Supplement: RA-008-C8RA04599B-s001 [file RA-008-C8RA04599B-s001.pdf]

Electronic Supplementary Material (ESI) for RSC Advances.
This journal is © The Royal Society of Chemistry 2018

Supplementary Information

Low temperature synthesis of $BiFeO_3$ nanoparticles with enhanced magnetization and promising photocatalytic performance in dye degradation and hydrogen evolution

M. A. Basith, Nilufar Yesmin, Rana Hossain
Department of Physics, Bangladesh University of Engineering and Technology, Dhaka-1000, Bangladesh

Table-S1: Rietveld Refined structural parameters and phases present (in wt %) obtained from powder XRD

Sample	Crystal Structure	Lattice Parameters (Å)	Atomic Coordinates	R Factor
BFO Bulk	BFO(R3c)<br>(Rhombohedral)<br>Fract (%): 79.79(0)<br>Vol: 373.370(0.004)	a=b=5.5764(2)<br>c=13.8640(1)	Bi (0,0,0)<br>Fe (0,0, 0.2202)<br>O(0.4478, 0.0006, 0.9545)	χ^2 = 2.30<br>R_p = 1.21<br>R_{wp} = 1.69
	$Bi_2Fe_4O_9$(pbam)<br>(Orthorhombic)<br>Fract(%): 8.69 (0)<br>Vol: 404.169(0.035)	a =8.4433(4)<br>b=7.9723(4)<br>c=6.0043(3)	Bi (0.17524, 0.17916, 0)<br>Fe1 (0.5, 0, 0.27910)<br>Fe2 (0.35714, 0.35660,0.5)<br>O1 (0, 0, 0.5)<br>O2(0.27003, 0.73403,-0.34894)<br>O3 (0.83303, 0.72656, 0.5)<br>O4(0.46749, 0.13604, 0.88808)	
	BFO(pnma)<br>(Orthorhombic)<br>Fract (%): 4.86(0)<br>Vol: 99.962(0.063)	a=3.6953(6)<br>b=4.1502(15)<br>c=6.517(3)	Bi (0.07672, -0.54738, -0.10374)<br>Fe (0.64829, 0.72185, 0.62450)<br>O1 (-0.29397, 0.39633, -0.14315)<br>O2 (0.26305, -0.27487, -0.34784)<br>O3 (0.08274, -0.34590, 0.07061)	
	$Bi_{25}FeO_{40}$ (I23)<br>(Cubic)<br>Fract(%): 6.66(0)<br>Vol: 1050.261(0.072)	a=b=c= 10.1648(4)	Bi (0.69420, -0.87266, 0.57740)<br>Bi/Fe (0,0,0)<br>O1(0.10396, 0.10328, 0.29397)<br>O2 (0.69383, -0.50582, 0.22787)<br>O3(0.75608, 0.75608, 0.75608)	
$Bi_{12.5}Fe_{0.5}O_{19.5}$ 120°C	$Bi_{12.5}Fe_{0.5}O_{19.5}$<br>(I23)<br>(Cubic)<br>Fract(%): 50.23(0.62)<br>Vol:1049.414(0.049)	a=b=c= 10.1620(3)	Bi1 (0.1782, 0.3149, 0.0139)<br>Fe (0,0,0)<br>Bi2 (-0.0310, -0.0310, -0.0310)<br>O1 (0.1336, 0.2364, 0.5141)<br>O2 (0.1694, 0.1694, 0.1694)<br>O3 (0.8937, 0.8937, 0.8937)	χ^2 =1.37<br>R_p =3.52<br>R_{wp} =4.82

	Bi_2O_3 (P121/c)<br>(Monoclinic)<br>Fract(%):<br>49.40(0.88)<br>Vol:<br>328.746(0.025)	a=5.8406(3)<br>b=8.1557(3)<br>c=7.4977(4)	Bi1 (0.5257, 0.1885, 0.6384)<br>Bi2 (0.0379, 0.0424, 0.2253)<br>O1 (0.6150, 0.2780, 0.3160)<br>O2 (0.6481, -0.0095, 0.0945)<br>O3 (0.0189, 0.0918, 0.4769)	
Bi_2O_3<br>140°C	$Bi_{12.5}Fe_{0.5}O_{19.5}$(I2<br>3)<br>(Cubic)<br>Fract(%):<br>29.98(0.32)<br>Vol:1050.125(0.0<br>36)	a=b=c=<br>10.1643(2)	Bi1 (0.1775, 0.3132, 0.0109)<br>Fe (0,0,0)<br>Bi2 -0.0205, -0.0205, -0.0205)<br>O1 (0.1257, 0.2617, 0.5488)<br>O2 (0.1778, 0.1778, 0.1778)<br>O3 (0.8892, 0.8892, 0.8892)	χ^2 =1.18<br>R_p =3.87<br>R_{wp} =4.92
	Bi_2O_3 (P121/c)<br>(Monoclinic)<br>Fract(%):<br>70.02(0.55)<br>Vol: 329.463(<br>0.020)	a=5.8443(2)<br>b=8.1618(2)<br>c=7.5053(3)	Bi1 (0.5296, 0.1835, 0.6368)<br>Bi2 (0.0418, 0.0421, 0.2201)<br>O1 (0.6377, 0.2910, 0.3843)<br>O2 (0.7170, -0.0619, 0.1571)<br>O3 (0.0189, 0.0918, 0.4769)	
BFO<br>160°C	BFO (R3c)<br>(Rhombohedral)<br>Fract(%): 100(0)<br>Vol:<br>373.327(0.014)	a = b =5.5758(1)<br>c =13.8656(4)	Bi (0,0,0)<br>Fe (0,0, 0.2215)<br>O (0.4598, 0.0184, 0.9519)	χ^2 =1.75<br>R_p =5.58<br>R_{wp} =7.27
BFO<br>180°C	BFO (R3c)<br>(Rhombohedral)<br>Fract(%):<br>98.24(0.01)<br>Vol:<br>373.314(0.01)	a = b =5.5761(1)<br>c =13.8634(3)	Bi (0,0,0)<br>Fe (0,0, 0.2198)<br>O (0.4694, 0.0247, 0.95)	χ^2 =1.54<br>R_p =5.42<br>R_{wp} =7.19
BFO<br>200°C	BFO(R3c)<br>(Rhombohedral)<br>Fract (%): 100(0)<br>Vol:<br>373.24(0.085)	a = b =5.5754 (1)<br>c =13.8645 (4)	Bi (0,0,0)<br>Fe (0,0, 0.2248)<br>O (0.4556, 0.0238, 0.9398)	χ^2 =1.93<br>R_p =5.85<br>R_{wp} =7.64
